# Immediate effects of short-term meditation on sensorimotor rhythm-based brain–computer interface performance

**DOI:** 10.3389/fnhum.2022.1019279

**Published:** 2022-12-20

**Authors:** Jeehyun Kim, Xiyuan Jiang, Dylan Forenzo, Yixuan Liu, Nancy Anderson, Carol M. Greco, Bin He

**Affiliations:** ^1^Department of Biomedical Engineering, Carnegie Mellon University, Pittsburgh, PA, United States; ^2^Department of Psychiatry, University of Pittsburgh, Pittsburgh, PA, United States

**Keywords:** brain–computer interface, BCI, sensorimotor rhythm, meditation, EEG

## Abstract

**Introduction:**

Meditation has been shown to enhance a user’s ability to control a sensorimotor rhythm (SMR)-based brain-computer interface (BCI). For example, prior work have demonstrated that long-term meditation practices and an 8-week mindfulness-based stress reduction (MBSR) training have positive behavioral and neurophysiological effects on SMR-based BCI. However, the effects of short-term meditation practice on SMR-based BCI control are still unknown.

**Methods:**

In this study, we investigated the immediate effects of a short, 20-minute meditation on SMR-based BCI control. Thirty-seven subjects performed several runs of one-dimensional cursor control tasks before and after two types of 20-minute interventions: a guided mindfulness meditation exercise and a recording of a narrator reading a journal article.

**Results:**

We found that there is no significant change in BCI performance and Electroencephalography (EEG) BCI control signal following either 20-minute intervention. Moreover, the change in BCI performance between the meditation group and the control group was found to be not significant.

**Discussion:**

The present results suggest that a longer period of meditation is needed to improve SMR-based BCI control.

## Introduction

Brain–computer interfaces (BCI) are powerful tools that allow direct interaction between a user’s central nervous system (CNS) and the outside world, bypassing the traditional neuromuscular pathway (Wolpaw et al., 2000; He et al., 2015, 2020). These systems have the potential to benefit millions, from those living with spinal cord injuries (SCI) and stroke to those with neurodegenerative diseases, such as amyotrophic lateral sclerosis (ALS) or multiple sclerosis (MS). One way of classifying BCIs is by their invasiveness, or whether they require surgical operations to import CNS data to a computer. Another way of classifying BCIs is by the source of the CNS input signal and their applications. One of the most widely utilized CNS signals is sensorimotor rhythm (SMR) produced in the primary sensorimotor areas during motor imagery (MI), or the imagination of movement (Pfurtscheller and Lopes da Silva, 1999; Yuan and He, 2014). Many previous studies have used SMR-based BCIs to move a computer cursor in 2D (Wolpaw and McFarland, 2004) and 3D (Meng et al., 2018), steer a powered wheelchair (Tanaka et al., 2005; Carlson and Millan, 2013; Yu et al., 2017), fly a virtual helicopter (Doud et al., 2011), fly a quadcopter (LaFleur et al., 2013), and control a robotic arm (Meng et al., 2016; Edelman et al., 2019), etc.

Despite the abundance and diversity of its applications, SMR-based BCI still faces many challenges. First of all, it has been reported that roughly 20% of the general population is known to be “BCI-inefficient,” meaning that they are unable to control a BCI system beyond chance level even with extensive training (Blankertz et al., 2010). The reason this subgroup of people is less successful at controlling a BCI system is still unknown. Another challenge with SMR-based BCI is that it requires a relatively long training time (He et al., 2020). Unlike exogenous BCIs that do not require a lot of training, such as those that use P300 (Eidel and Kübler, 2020) or steady-state visually-evoked potentials (SSVEP) (Chen et al., 2015), SMR-based BCI, which operates using users’ volitional CNS signals as input, exhibits a learning curve and requires several sessions of training before users can achieve good performance (Jiang et al., 2021b; Stieger et al., 2021b).

To address these challenges, recent efforts have focused on improving both facets of an SMR-based BCI. On the one hand, a great deal of research has focused on improving the signal processing and decoding aspect of SMR-based BCI, i.e., the computer side of a BCI system (Bashashati et al., 2007). Recent advancements in machine learning (ML) and deep learning (DL) have made progress in the field of BCI as well. Previous works have examined the potential of using convolutional neural networks (Schirrmeister et al., 2017; Lawhern et al., 2018; Stieger et al., 2021a; Zhu et al., 2022), deep belief networks (Ren and Wu, 2014; Lu et al., 2017), and recurrent neural networks (Luo et al., 2018; Ma et al., 2018; Wang et al., 2018) to improve MI classification. On the other end of the spectrum are studies that explore ways to train humans to improve BCI performance, one of which methods is via meditation. For example, Mokhtar et al. showed that it is possible to measure the level of attention and meditation using Electroencephalography (EEG) (Mokhtar et al., 2017). Further, meditation in varying forms has been reported to facilitate BCI learning. Cassady et al. found that those with one or more years of experience in mind-body awareness training (MBAT), such as yoga and meditation, not only improved BCI accuracy more quickly, but also improved to a significantly greater extent compared to those without prior experience (Cassady et al., 2014). Tan et al. reported that a 12-week mindfulness meditation training significantly improved SMR-based BCI accuracy compared to a music training group and a control group (Tan et al., 2014). More recently, Stieger et al. showed that taking an 8-week mindfulness-based stress reduction (MBSR) course results in faster BCI learning compared to a control group (Stieger et al., 2021b). While there is no consensus on the exact mechanism behind how meditation in its various forms positively affects BCI learning, researchers in these prior works conjecture that mindfulness meditation helps with regulating emotions, stress, and attention (Tan et al., 2014), that MBAT helps with modulating the mu rhythm and thus improves concentration (Cassady et al., 2014), and that MBSR helps actively up-regulate the alpha power during volitional rest (Stieger et al., 2021b) and increases frontolimbic alpha activity (Jiang et al., 2021a).

While mindfulness meditation training, e.g., an 8-week MBSR course or yoga, is beneficial in expediting BCI learning, such training is time-consuming and requires guidance from experienced meditation instructors (Irving et al., 2009); thus, it is not an ideal option to enhance BCI performance for those requiring the use of an SMR-based BCI immediately (or within a very short period). Therefore, it is of interest to investigate the potential immediate effects of short-term meditation in a form that can be easily practiced by those that are unable to participate in active and regular meditation courses, such as taking routine yoga classes or enrolling in an 8-week MBSR course. As such, the motivation of the present study is to examine if a brief mindfulness meditation practice would have an immediate effect on SMR-BCI performance. A previous study (Stieger et al., 2021b) found that MBSR training can significantly improve the ability to modulate alpha rhythm as associated with the up-down (UD) task. In this study, we examined if a 20-min mindfulness exercise would affect the level of mindfulness and thus BCI performance in the UD task. We also examined if the change in BCI performance following two types of intervention is different, i.e., a brief meditation exercise induces different effects on the brain compared to a control exercise.

## Materials and methods

### Participants

Thirty-seven subjects (13 male, 22 female, 2 non-binary) were recruited via a mass email and physical fliers across the Carnegie Mellon University campus in Pittsburgh, Pennsylvania. All subjects provided written informed consent to the study, which was approved by the Institutional Review Board of Carnegie Mellon University. No subject had prior experience using an SMR-based BCI. In terms of mindfulness meditation experience, all subjects met the following inclusion criteria: no mindfulness meditation experience at all, or only having non-regular meditation experience that happened more than 6 months prior to the date of the first session. Mindfulness is operationally defined as non-judgmental awareness and acceptance of the present thoughts, feelings, and sensations (Bishop et al., 2004), and meditation can be defined as a quiet or audio-guided period of contemplation that can cultivate one’s capacity for mindfulness. Of the 37 subjects, 10 were assigned to group MM (meditation-meditation group, age = 21.9 ± 3.1), 12 were assigned to group MC (meditation-control group, age = 24.7 ± 4.4), and 15 were assigned to group CM (control-meditation group, age = 21.7 ± 3.7). Group CC was not included because the comparisons among MM, MC, and CM are already sufficient to address the research question in this study. Those assigned to group MM were asked to listen to and follow along the guided meditation exercise as the intervention in both sessions. Those in group MC were asked to listen to and follow along the guided meditation exercise in the first session but listen to the control recording—a journal paper—in the second session. Those in group CM listened to the control recording in the first session and the guided meditation recording in the second session. One subject from group MC did not complete the second session and thus was dropped from behavioral analysis of session 2. In the EEG neurophysiological analysis, a total of 6 subjects were excluded: from group MC, 1 subject was excluded due to equipment issues and 1 other subject for not completing the study (the same subject as before); and from group CM, 1 subject was excluded due to equipment issues, 1 subject due to experimental error, and 2 subjects due to their EEG data containing too many artifacts to perform the offline analysis.

### Experimental design

**FIGURE 1:**
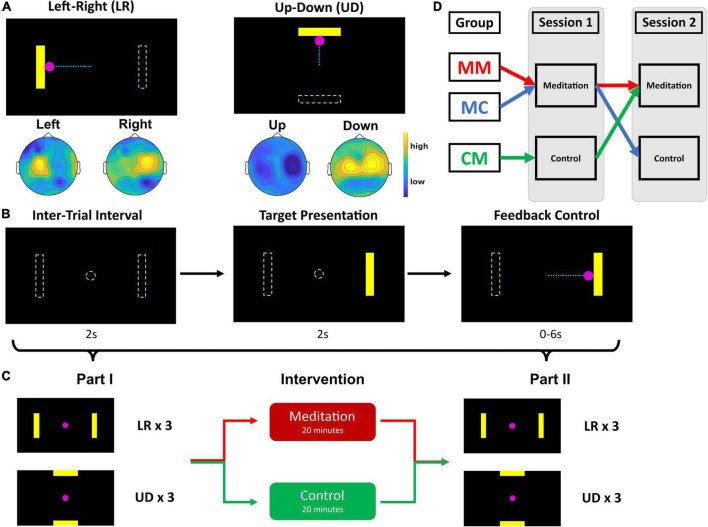
Schematic diagram of study design. **(A)** BCI tasks. Left-right (LR) and up-down (UD), the dashed lines are for illustrative purposes only and were not visible in the actual experiment. Scalp topographies show regions of mu power increase (yellow) and decrease (blue) during each trial type (i.e., left vs right and up vs down). The colors represent the distribution of mu rhythm power calculated for each electrode during the different trial types. **(B)** Each trial: 2 s of intertrial interval, 2 s of target presentation, and up to 6 s of cursor control with visual feedback. **(C)** Session design: Each session consists of part 1, intervention, and part 2. Part 1 contains 3 runs of LR and 3 runs of UD, where one run consists of 25 trials of BCI control. Intervention is either a 20-min meditation or control task, and part 2 contains 3 runs of LR and 3 runs of UD tasks each (practice runs for the LR and UD tasks in session 1 are not included in the figure). **(D)** Group assignment: Group MM (*N* = 10) performed brief meditation with audial guidance in both sessions; group MC (*N* = 12) performed meditation with audial guidance in session 1 and listened to control recording in session 2; group CM (*N* = 15) listened to control recording in session 1 and performed brief meditation with audial guidance in session 2.

The experimental design is illustrated in Figure 1. This study consisted of two sessions on two separate days, anywhere from 2 to 28 days apart depending on subject availability. The two sessions were separated by a minimum of 2 days to reduce the possible carryover effect of the 20-min intervention in session 1. Each session consists of part 1, followed by a 20-min intervention, and then part 2 (Figure 1D). Session 1 part 1 contains 8 runs: 4 runs of a one-dimensional (1D) left-right (LR) cursor control task and 4 runs of a 1D up-down (UD) cursor control task (Figure 1A), of which the first run in both tasks were practice runs. The purpose of adding these practice runs was to familiarize the subjects with the BCI tasks. Data from these two runs were not used for further analysis. Given that prior work shows BCI learning can occur even after 20–30 runs (Jiang et al., 2021b), discarding the very first run will have a limited effect on studying the BCI learning in this work, and we can avoid potentially inaccurate BCI data when the subjects are just starting to learn the BCI task. Thus, only 6 runs of BCI data were saved from session 1 part 1.

Based on their performance in these 6 runs—excluding two practice runs—the subjects were assigned to one of three groups: group MM, for meditation in both sessions; group MC, for meditation in session 1 and control exercise in session 2; and group CM, for control exercise in session 1 and meditation in session 2. Group assignment was conducted with the intention of maintaining similar group average performance levels in session 1 part 1 so that the change in group BCI performance average would be directly comparable among the groups.

Once the subjects were assigned groups, they were instructed to practice meditation with a 20-min audio recording of a guided meditation exercise (meditation) or listen to a journal paper about mindfulness and its Buddhist origins (control) (Purser and Milillo, 2015). Immediately following the intervention, the subjects were instructed to fill out the Toronto Mindfulness Survey (TMS), a 13-item survey that measures the transient level of mindfulness (Lau et al., 2006).

Afterward, in session 1 part 2, subjects repeated the same 6 runs of the BCI tasks as before the intervention—3 runs of LR followed by 3 runs of UD (Figure 1C). Session 2 parts 1 and 2 were identical to session 1 part 2—3 runs each of LR and UD. As for the interventions in session 2, those in group MM and group CM performed brief meditation exercises following audio guidance, whereas those in group MC listened to the recording of the journal paper (control recording). By creating two cohorts of subjects that participated in different interventions, we were able to control for within-person effects of continued use of an SMR-based BCI, such as learning and fatigue, and only investigate the effects of meditation.

### Brain–computer interface tasks

Figures 1A–C illustrates the standard BCI2000 cursor control tasks (Schalk et al., 2004) used in this study. In each experimental session, subjects sat in a chair with armrests in front of a computer monitor. The monitor initially would display a black screen with the word “Timeout” at the center. Once the experimenter started a run, a yellow rectangular bar would appear either on the left or right (for the LR task), or on the top or bottom (for the UD task) side of the screen. 2 s after this “target” appeared, a pink circle, or the “cursor,” appeared at the center of the monitor (Figure 1B). The purpose of the tasks was for the subjects to move the cursor toward the target and hit it. If the cursor hit the correct target on the screen within 6 s, it would change colors from pink to yellow to visually indicate that the trial was successful. If the subject was not able to hit the target with the cursor within the given 6 s, the trial ended and moved on to the next trial. If the cursor moved in the opposite direction of the target and hit the invisible target on the other side, the trial would end and be recorded as a miss.

To move the cursor either to the left or the right, the subjects were instructed to perform MI with the corresponding hand. For example, if the target appears on the right side of the screen, the subjects would perform right-hand MI. In the UD task, subjects were instructed to perform MI with both hands simultaneously to move the cursor up and voluntarily rest and not perform MI to move the cursor down. Each trial would last either until the cursor hit the correct (“hit”) or incorrect (“miss”) target, or for 6 s, whichever is shorter. Following the end of each trial were 2 s of inter-trial intervals and each run consisted of 25 trials.

To extract the control signal for online cursor movement, a small surface Laplacian filter was first applied to the C3 and C4 electrodes, from which the autoregressive (AR) spectral amplitudes were estimated in a 3 Hz bin (Stieger et al., 2021b) around 12 Hz (Meng et al., 2016, 2018). The main advantage of using a surface Laplacian filter is that it improves spatial resolution (Carvalhaes and de Barros, 2015) by enhancing local cortical activity while reducing activity from distant sources, such as muscle or motion artifacts and eye movements (Mourino et al., 2001). AR estimation of spectral amplitudes, which is based on the principle of maximum entropy method (MEM), is preferred over fast Fourier transform (FFT), another spectral analysis method, due to its support of high-resolution spectral analysis with short time segments (McFarland et al., 1997) and computational efficiency (He et al., 2020), allowing for quick calculations and output signal generation. Then, the control signal was normalized to a zero mean and unit variance by subtracting an offset and multiplying a gain value which was estimated from a buffer of 30 s, which reduces the influence of abrupt abnormal EEG on the control signal and makes it relatively smooth during BCI control. The cursor speed was determined by the normalized AR amplitude difference and the cursor position was updated every 40 ms. For horizontal motion (LR tasks), the control signal was produced by taking the difference in AR amplitude between the two electrodes (C4 - C3), and for vertical motion (UD tasks), the signal was produced by taking the sum of the AR amplitudes of the two electrodes (C4 + C3). Each trial had three possible outcomes: a “hit” when the cursor successfully hit the target within 6 s and changed colors; a “miss” when the cursor hit the invisible target on the opposite side from where the target appeared; and an “invalid” when the subject was unable to hit the target with the cursor within 6 s. BCI performance, or MI accuracy, was quantified using percent valid correct (PVC) (Doud et al., 2011; Cassady et al., 2014; Meng et al., 2016; Edelman et al., 2019; Jiang et al., 2021b), which is the proportion of the number of “hits” within the total number of “hits” and “misses.”

### Electroencephalography acquisition and preprocessing

EEG data from the subjects were acquired using the Neuroscan SynAmps system with the 64-channel EEG QuikCap (Neuroscan Inc., Charlotte, NC) according to the extended 10–20 system. The EEG data were sampled at a frequency of 1,000 Hz and filtered between 0.1 and 100 Hz, with an additional notch filter at 60 Hz to remove powerline noise. The impedance of each electrode was kept below 5 kΩ during preparation.

Each session started by recording 5 min of resting state EEG data. During these 5 min, subjects were instructed to sit still with their eyes open and refrain from making large or sudden motions while EEG was collected. After that, the EEG during BCI tasks was also collected.

The EEG data were preprocessed according to the following steps using the MATLAB toolboxes EEGLAB (version 2021.1) and FieldTrip (version 20220714). First, bad channels were visually inspected and rejected based on the EEG data variance using FieldTrip. If a channel was rejected, the data of that channel were spherically interpolated. The data were also bandpass filtered between 1 and 100 Hz and downsampled to 250 Hz. Next, it was re-referenced using common average reference (CAR), and independent component analysis (ICA) was used to extract and remove artifacts, including but not limited to eye blinks and other motion artifacts. Independent components (IC) were removed if they were deemed to be motion artifacts. Lastly, the data were corrected for baseline drift and subsequently linearly detrended. For EEG analysis of BCI, certain trials with abnormally large variance were also rejected.

### Audio recordings for intervention

The two intervention recordings were both approximately 20 min long and recorded by a yoga instructor. The guided mindfulness meditation exercise recording aimed to calm the listener and direct them to focus their attention on their bodies and their surroundings. In the beginning, there was a short exercise where the subjects were instructed to count their breaths. Breath-counting training was included because it is known to improve mindfulness and decrease mind wandering (Levinson et al., 2014). Approximately 10 min of the 20 min were spent imagining parts of the body moving. The control recording, on the other hand, is simply a voice recording of the narrator reading out loud the journal article: *Mindfulness Revisited: A Buddhist-Based Conceptualization* (Purser and Milillo, 2015). This manuscript was chosen because while it is a manuscript on a similar topic—mindfulness—it does not actually guide the listener through a meditation session. Due to limited time, however, the reader was only able to read the first few pages of the article. Throughout the 20 min of both interventions, the subjects’ EEG data were recorded.

We tried our best to ensure that the subjects were paying attention and indeed following the instructions. For example, before starting the audio files, we instructed them to not simply listen but really engage and follow the instructions, such as focusing their minds on different parts of their bodies, imagining moving certain joints, etc. Further, as a way of keeping them focused, we also reminded subjects of how much time they had left at 10 and 15 min into the interventions. In our opinion, this was the best we could do to ensure their engagement with the mindfulness intervention.

### Measure of mindfulness

The Toronto Mindfulness Scale (TMS) was used to measure the current, transient level of mindfulness of the subjects (Lau et al., 2006) following the 20-min interventions. The TMS asks the respondent to look back at “what you just experienced” to answer a set of 13 statements that “people sometimes experience” on a 5-point scale from “not at all” to “very much.” The statements are divided into two subscales–the Curiosity score and the Decentering score–and for each subscale and TMS as a whole, a higher score implies a higher level of mindfulness.

In this particular study, the TMS was used in lieu of the Freiburg Mindfulness Inventory (FMI) and the Mindfulness Attention Awareness Scale (MAAS), which were used in a previous study (Jiang et al., 2021b). Unlike the TMS, the FMI and the MAAS ask the respondent to look back at the past few days to answer the questions (Brown and Ryan, 2003; Walach et al., 2006), thus rendering these surveys inappropriate for this study. Because we wished to investigate the change in mindfulness following a brief, 20-min intervention, we opted for the TMS.

### Statistical analysis

The statistical tests that were used in this work are the Wilcoxon signed-rank (WSR) test, the Wilcoxon rank-sum (WRS) test, and Spearman’s rank correlation. The WSR test is used to test the statistical difference across two interventions in the same subject population. Therefore, this test was used to compare the TMS scores of those that participated in both interventions (groups MC and CM) and the change in EEG data and BCI performance following each intervention in the two groups. The WRS test is used to compare the effects of two interventions in two separate groups. Thus, this test was used to compare the change in BCI performance across groups that participated in different interventions for each session, i.e., groups MM and MC compared with group CM in session 1, and group MM compared with group MC in session 2. Lastly, Spearman’s rank correlation was used to investigate the association between TMS scores and BCI performance, and the association between change in BCI performance and the number of days between the two sessions. Nonparametric tests were used during statistical analysis because the data collected did not show a Gaussian distribution, and for certain tests, the sample size for the groups was not deemed large enough to warrant the use of a parametric test. All statistical tests were conducted in RStudio (1.4.1103).

## Results

### Mindfulness survey scores

The scores of the mindfulness survey taken immediately after the interventions were compared and are summarized in Table 1. First, those in group MM (*n* = 10)–meditation as the intervention in both sessions–did not show a significant change in the TMS scores, as was predicted. The group average total TMS score following the meditation exercise in session 1 is 32.6 ± 10.38, whereas the score following the meditation exercise in session 2 is 31.6 ± 13.48 (WSR, *Z* = −0.21, *p* = 0.84). Next, we compared the TMS scores among subjects that participated in both interventions, i.e., those in group MC (*n* = 11, meditation in session 1 and control in session 2) and group CM (*n* = 15, control in session 1 and meditation in session 2). Overall, we did not find a significant difference in the TMS scores between the two interventions. In group MC, the average total TMS score following the meditation exercise in session 1 is 32.91 ± 9.42, whereas the average score following the control exercise in session 2 is 28.55 ± 11.47 (WSR, *Z* = −1.47, *p* = 0.14). Those in group CM also did not show a significant change in the total TMS score. The group average total TMS score following the control exercise in session 1 is 29.2 ± 7.12, whereas the score following the meditation exercise in session 2 is 28.2 ± 12.37 (WSR, *Z* = −0.07, *p* = 0.94).

**TABLE 1:** Group average Toronto Mindfulness Survey and subscale scores for each session (mean ± stdev).

Group	(Sub)score	Session 1	Session 2	*p*
MM	Total	32.6 ± 10.38	31.6 ± 13.48	0.84
	Curiosity	15.9 ± 5.47	15 ± 6.46	0.43
	Decentering	16.7 ± 6.48	16.6 ± 7.49	0.86
MC	Total	32.91 ± 9.42	28.55 ± 11.47	0.14
	Curiosity	15.18 ± 6.29	13 ± 6.69	0.10
	Decentering	17.73 ± 4.31	15.55 ± 6.09	0.33
CM	Total	29.2 ± 7.12	28.2 ± 12.37	0.94
	Curiosity	15.4 ± 6.73	12.8 ± 7.78	0.16
	Decentering	13.8 ± 4.41	15.4 ± 6.63	0.15

*P*-values compare the group average scores between the two sessions. The meditation exercise was played in session 1 for groups MM and MC and in session 2 for group CM. The control exercise was played in session 2 for groups MM and MC and in session 1 for group CM.

When we separated the TMS statements into the two subscales, we saw a greater difference in the subscale scores between the intervention types. Again, the subscale scores from group MM remain unchanged. The Curiosity subscale score of the meditation exercise in session 1 is 15.9 ± 5.47 and that of the meditation exercise in session 2 is 15 ± 6.46 (WSR, *Z* = −0.79, *p* = 0.43). The Decentering subscale score of the meditation exercise in session 1 is 16.7 ± 6.48 and that of the meditation exercise in session 2 is 16.6 ± 7.49 (WSR, *Z* = −0.18, *p* = 0.86). For group MC, the Curiosity subscale score of the meditation exercise in session 1 is 15.18 ± 6.29 and that of the control exercise in session 2 is 13 ± 6.69 (WSR, *Z* = −1.64, *p* = 0.10). The Decentering subscale score of the meditation exercise in session 1 is 17.73 ± 4.31, whereas that of the control exercise in session 2 is 15.55 ± 6.09 (WSR, *Z* = −0.98, *p* = 0.33). Lastly, for group CM, the Curiosity subscale score of the control exercise in session 1 is 15.4 ± 6.73 and that of the meditation exercise in session 2 is 12.8 ± 7.78 (WSR, *Z* = −1.41, *p* = 0.16). The Decentering subscale score of the control exercise in session 1 is 13.8 ± 4.41, whereas that of the meditation exercise in session 2 is 15.4 ± 6.63 (WSR, *Z* = −1.45, *p* = 0.15).

### Toronto Mindfulness Survey scores and brain–computer interface performance

First, we examined the relationship between the TMS scores, regardless of intervention type, and overall BCI performance in the UD task following the interventions. We investigated the correlation between the TMS scores and post-intervention BCI performance only because we believed that the TMS scores represented the subjects’ level of mindfulness at that moment when they were just about to start part 2 of each session. The Spearman correlation coefficients showed no significant association between the TMS scores and post-intervention BCI performance. The Spearman correlation coefficient between the total TMS score and BCI performance in the UD task following intervention is 0.076 (*p* = 0.53). The Spearman correlation coefficient between the Curiosity subscale score and UD BCI performance is 0.013 (*p* = 0.92), and that between the Decentering subscale score and UD BCI performance is 0.060 (*p* = 0.62). For comparison, the Spearman correlation coefficient between the total TMS, the Curiosity subscale, and the Decentering subscale scores, and the LR task performance are 0.146 (*p* = 0.23), 0.151 (*p* = 0.21), and 0.043 (*p* = 0.72), respectively.

Then, we wanted to determine whether the level of mindfulness is related to a change in BCI performance in the UD task. To do this, we calculated the Spearman correlation coefficients between the total TMS scores, along with its two subscales, and the change in UD PVC. For those that completed the meditation intervention, the Spearman correlation coefficients between the total TMS score, the Curiosity subscore, and the Decentering subscore, and the change in UD PVC are 0.146 (*p* = 0.34), 0.075 (*p* = 0.63), and 0.170 (*p* = 0.26), respectively. The correlation coefficients for those that completed the control intervention are 0.155 (*p* = 0.46), −0.012 (*p* = 0.96), and 0.251 (*p* = 0.23), respectively. For comparison, the correlation coefficients for the LR task are 0.117 (*p* = 0.44), 0.160 (*p* = 0.29), and 0.041 (*p* = 0.79) for the meditation intervention, and −0.058 (*p* = 0.78), −0.038 (*p* = 0.86), and −0.074 (*p* = 0.73) for the control intervention.

### Comparing the EEG power during meditation, control, and resting state

Next, we compared the EEG power during the meditation and control interventions and during resting state. Only those that completed both interventions (meditation and control) were considered in this analysis, i.e., those in groups MC and CM. A total of 21 subjects (10 from group MC and 11 from group CM) were included in this group analysis.

**FIGURE 2:**
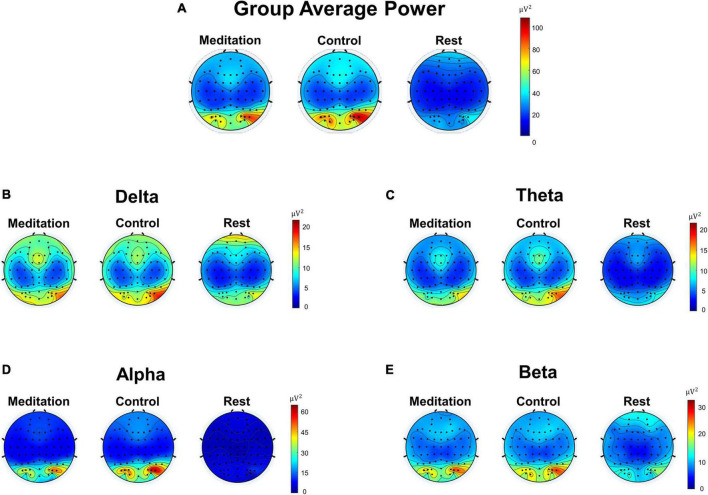
Scalp topographic maps of group (*n* = 21) average Electroencephalography (EEG) power of different frequency bands during meditation and control interventions. **(A)** Pre-processed average EEG power not filtered to any specific frequency band (1–100 Hz). **(B)** Average delta band (1–4 Hz) EEG power. **(C)** Average theta band (4–7 Hz) EEG power. **(D)** Average alpha band (8–12 Hz) EEG power. **(E)** Average beta band (13–30 Hz) EEG power.

Figure 2 shows the overall group average of EEG power and EEG power bandpassed to four frequency bands: delta (1–4 Hz), theta (4–7 Hz), alpha (8–12 Hz), and beta (13–30 Hz). Figure 2A shows the average EEG power of each electrode, calculated by taking the average of the squared difference between the raw EEG value at each time point and the mean EEG of that electrode. The group average was calculated by taking the average EEG power of each electrode for all 21 subjects. We see electrodes with high power in the occipital region during meditation and control. In contrast, the resting state EEG presents minimal power compared to meditation and control. Figures 2B–E shows the group average power of EEG bandpassed to each frequency band: delta, theta, alpha, and beta. Again, we see very similar EEG patterns between meditation and control states, whereas the resting state shows very low power in all frequency bands.

### Comparing the change in brain–computer interface performance following each intervention

**FIGURE 3:**
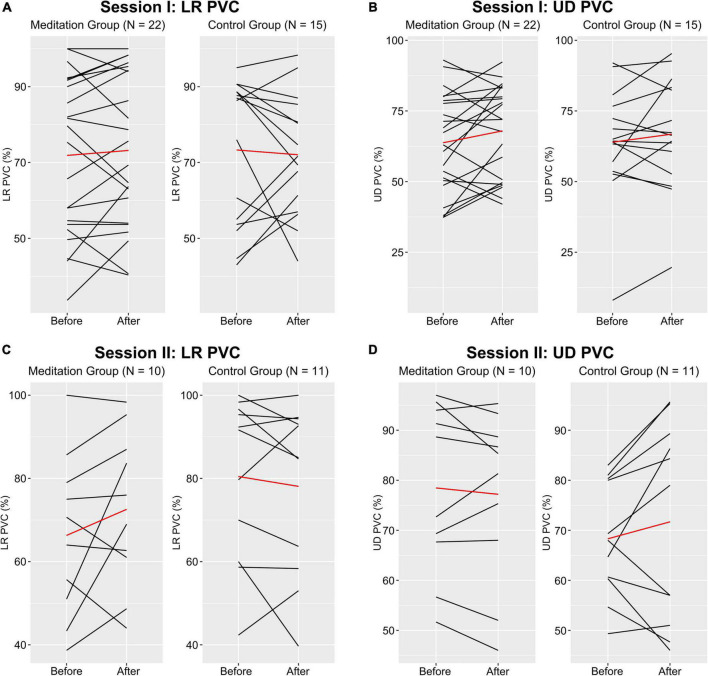
Line graphs of each subject’s performance in the LR and UD tasks in both sessions. Black lines represent each subject’s average LR and UD PVC before and after each intervention and the red lines represent the group average PVC. **(A)** Change in LR PVC for meditation group (group MM and group MC) and control group (group CM) from session 1. **(B)** Change in UD PVC for meditation group and control group from session 1. **(C)** Change in LR PVC for meditation group and control group from session 2. **(D)** Change in UD PVC for meditation group (group MM) and control group (group MC) from session 2.

To investigate the effect of the two types of intervention on BCI performance, we compared the change in Percent Valid Correct (PVC)—the ratio between the number of “hits” and the sum of “hits” and “misses”—between the group of subjects that participated in the meditation exercise and those that participated in the control exercise (Figure 3). In session 1, we compared the change in PVC following the interventions between group M (*n* = 22, group MM and group MC) and group CM (*n* = 15). Overall, neither intervention seemed to have a significant effect on BCI performance. The meditation subjects (group M) saw a change in LR PVC of 1.30 ± 9.42% (WSR, *Z* = −0.69, *p* = 0.49) and the control subjects (group CM) saw a change in LR PVC of −1.27 ± 14.40% (WSR, *Z* = −0.14, *p* = 0.89). In the UD task, the meditation subjects (group M) saw a PVC change of 4.20 ± 11.82% (WSR, *Z* = −1.48, *p* = 0.14) and the control subjects (group CM) saw a PVC change of 2.84 ± 11.04% (WSR, *Z* = −0.77, *p* = 0.44). Here, a positive change in PVC refers to an improvement in BCI performance while a negative change in PVC refers to a deterioration in BCI performance. Although there seems to be a numerical increase in UD PVC following the meditation exercise–which is in line with previous report (Stieger et al., 2021b)– this effect disappears in session 2.

In session 2, we compared the change in PVC between group MM (*n* = 10) and group MC (*n* = 11). One subject in group MC did not complete session 2 and was dropped from this part of the analysis. Again, we did not find a significant effect on BCI performance from either intervention. The subjects that participated in the meditation exercise (group MM) saw a change in LR PVC of 6.27 ± 14.24% (WSR, *Z* = −0.82, *p* = 0.41) and the control subjects (group MC) saw a change in LR PVC of −2.36 ± 9.60% (WSR, *Z* = −0.67, *p* = 0.50). Likewise, the meditation subjects (group MM) saw a UD PVC change of −1.27 ± 5.60% (WSR, *Z* = −0.71, *p* = 0.48) and the control subjects (group MC) saw a UD PVC change of 3.39 ± 11.38% (WSR, *Z* = −0.93, *p* = 0.35). The reason for only comparing the subjects in these two groups is that it may be inappropriate to directly compare the subjects across groups M (groups MM and MC) and CM because the subjects participated in different interventions in session 1. This was also the reason for splitting the meditation group (group M) into two smaller groups (group MM and group MC). In contrast, directly comparing the subjects in groups MM and MC is acceptable because they come from the same cohort of meditators in session 1. For comparison, group CM saw a change in LR PVC of 3.76 ± 16.56% (WSR, *Z* = −0.19, *p* = 0.85) and a change in UD PVC of 0.09 ± 11.10% (WSR, *Z* = −0.50, *p* = 0.62).

### Comparing the change in brain–computer interface performance across groups

Despite the non-significant change in BCI performance following the two interventions, we can still examine the difference in the PVC change between the two groups in both sessions. In session 1, the meditation subjects (group MM and group MC) saw a change in LR PVC of 1.30 ± 9.42% and the control subjects (group CM) saw a change in LR PVC of −1.27 ± 14.40% (WRS, *Z* = −0.34, *p* = 0.73). In the UD task, the meditation subjects (group MM and group MC) saw a PVC change of 4.20 ± 11.82% and the control subjects (group CM) saw a PVC change of 2.84 ± 11.04% (WRS, *Z* = −0.34, *p* = 0.73). This outcome is repeated in the second session. In session 2, the subjects that participated in the meditation exercise (group MM) saw a change in LR PVC of 6.27 ± 14.24% and the control subjects (group MC) saw a change in LR PVC of −2.36 ± 9.60% (WRS, *Z* = −1.02, *p* = 0.31). In the UD task, the meditation subjects (group MM) saw a UD PVC change of −1.27 ± 5.60%, and the control subjects (group MC) saw a UD PVC change of 3.39 ± 11.38% (WRS, *Z* = −1.09, *p* = 0.28). In short, neither intervention significantly affected BCI performance in either BCI task (LR or UD), and the difference in the change in BCI performance between the interventions was also not found to be significant.

### Association between change in brain–computer interface performance and interval between sessions

**FIGURE 4:**
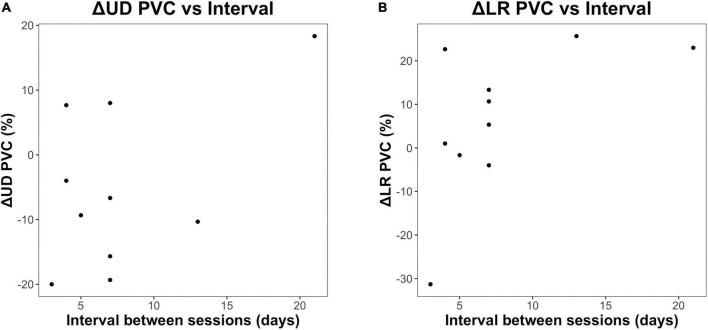
Scatter plots of difference in change in BCI performance with respect to the interval between sessions in number of days. **(A)** Difference in the change in LR PVC across the two sessions with respect to the interval between sessions. **(B)** Difference in the change in UD PVC across the two sessions with respect to the interval between sessions.

While the main purpose of this study was to investigate the immediate effect of a short, 20-min mindfulness meditation on SMR-based BCI performance, we also explored whether the interval between the two sessions (in days) was associated with a change in BCI performance (PVC, %) following meditation for group MM (*n* = 10). Only group MM was considered for this analysis because they performed the same intervention (mindfulness meditation) in both sessions, and it made sense to compare whether the change in BCI performance across the two sessions was significantly different. The plots (Figure 4) seem to show a general increase in performance improvement as the interval between sessions increases, with the Spearman correlation coefficients being 0.634 for the LR (*p* = 0.049) and 0.276 for the UD task (*p* = 0.440), respectively. However, given the small number of subjects and large variability in the data, it is not rigorous to conclude that the interval between sessions is strongly correlated with BCI performance.

### EEG during brain–computer interface

We calculated the offline control signal that was as close to the real control signal used for the online BCI2000 task. First, we calculated the small Laplacian filtered C4 and C3 mu rhythm power. Then, for the LR task, the offline control signal was defined as C4 power minus C3 power (C4 - C3), and for the UD task, the offline control signal was defined as C4 power plus C3 power (C4 + C3). After that, this offline control signal was normalized to zero mean and unit variance. Finally, we defined and computed the quantity △control signal as the difference in offline control signal between the left and right trials in the LR task, and between the up and down trials in the UD task (Jiang et al., 2021b).

**FIGURE 5:**
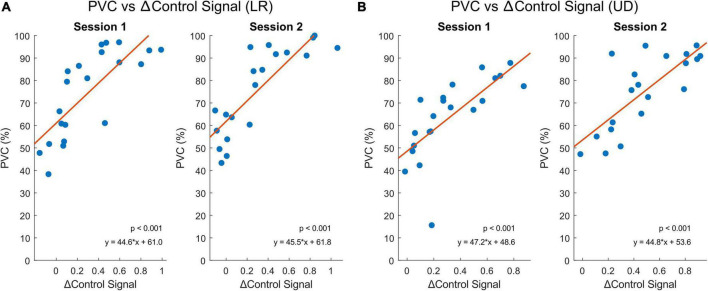
The relationship between △control signal and BCI performance (PVC, %) from both sessions using Spearman’s correlation. Session 1 contains subjects from group MC (*n* = 10) and group CM (*n* = 11) and session 2 contains subjects from group MM (*n* = 10) and group MC (*n* = 10). Blue dots represent each subject and orange lines represent the linear regression models of the individual points. **(A)** BCI performance correlates significantly with the △control signal between the left and right trials (*p* < 0.001). **(B)** BCI performance correlates significantly with the △control signal between the up and down trials (*p* < 0.001).

Theoretically, the larger the △control signal, the better the performance should be, since the greater the EEG power difference between C4 and C3, the easier it is to classify between left and right, and up and down. We indeed observed a strong and positive correlation between the PVC and the trial-averaged △control signal for LR and UD tasks (*p* <0.001), as shown in Figure 5. Session 1 compares group MC (*n* = 10) to group CM (*n* = 11), and session 2 compares group MM (*n* = 10) to group MC (*n* = 10).

**FIGURE 6:**
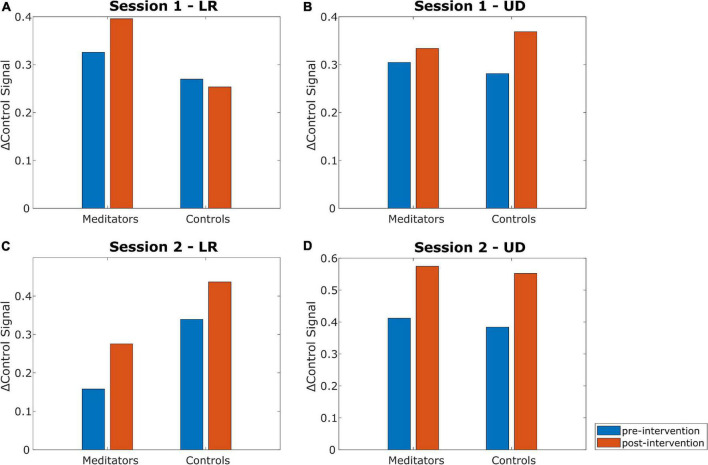
Changes in the △control signal for the LR and UD tasks in both sessions. Session 1 meditators are from group MC (*n* = 10) and control subjects are from group CM (*n* = 11). Session 2 meditators are from group MM (*n* = 10) and control subjects are from group MC (*n* = 10). Within-session changes and inter-group differences were analyzed using the Wilcoxon signed-rank and Wilcoxon rank-sum test, respectively. None of the comparisons (pre- vs. post-intervention, meditators vs. controls) showed statistically significant difference. **(A)** Changes in the △control signal for the LR task in session 1. **(B)** Changes in the △control signal for the UD task in session 1. **(C)** Changes in the △control signal for the LR task in session 2. **(D)** Changes in the △control signal for the UD task in session 2.

Next, we looked at how the △control signal changed from pre- to post-intervention and compared between the two intervention types. Figure 6 summarizes the results. Statistical analysis was performed using the Wilcoxon signed-rank test to compare within-session changes (pre- vs post-intervention) and the Wilcoxon rank-sum test to compare the differences in change of △control signal from pre- to post-intervention between the two groups (meditation vs control). There did not seem to be a trend with respect to which intervention caused a greater change in the △control signal. For example, meditators seemed to improve in the LR control signal in both sessions, whereas control subjects seemed to improve in only one of them. Similarly, control subjects seemed to improve in the UD control signal in both sessions, whereas meditators seemed to improve in only one of them. None of the tests (pre- vs post-intervention or meditators vs controls) in any session or task (LR and UD) showed a *p*-value less than 0.10.

## Discussion

Previous studies have investigated the effects of long-term meditation and an 8-week MBSR training course on SMR-based BCI performance. Results suggest that MBAT, in the forms of yoga and MBSR, has a positive impact on BCI learning (Cassady et al., 2014; Stieger et al., 2021b). To further explore the effects of a quick and easy MBAT exercise, in this study, we investigated the immediate effects of a 20-min meditation exercise on SMR-based BCI performance. The results show that a short mindfulness exercise does not significantly affect BCI performance in 1D cursor control tasks.

First, we examined the effect of a short, 20-min mindfulness exercise on the quantitative level of mindfulness as measured by the TMS. The results show that while there seems to be a numerical difference in the Decentering subscale within the TMS between the two intervention types, the effect is not significant. The creators of the TMS validated the legitimacy of using the TMS to measure and compare the level of mindfulness between those with either zero or at least 8 weeks of mindfulness meditation experience (Lau et al., 2006). In contrast, the subjects recruited for this study either had no experience in the past 6 months or do not consider themselves regular meditators. Therefore, the ability to evoke a certain level of mindfulness may be lacking for the subjects in this study. While it is encouraging that the 20 min of mindfulness exercise was able to produce a numerically higher Decentering subscale score compared to the control exercise, whether a longer or more frequent meditation will lead to a statistically significant difference needs to be studied. Another possible explanation is that the specific meditation intervention used in this study was not able to sufficiently impact the subjects to the degree where it would improve BCI performance. Future work investigating whether different types of meditation interventions, and whether a longer duration (between 20 min and 8 weeks) of meditation affects BCI performance would help clarify this ambiguity.

Further, it is interesting to note that there was a larger difference in the Decentering subscale than the Curiosity subscale between the two interventions. According to the creators of the TMS, the Curiosity subscale contains statements that reflect the awareness of the present experience, whereas the Decentering subscale focuses on the ability to distance from one’s thoughts and feelings and openness to experience (Lau et al., 2006). Hence, we predicted that because meditation is the awareness of the present moment (Schmidt and Walach, 2014), if there were to be a difference in the subscale scores, it would be in the Curiosity subscale. We believe that the control exercise, where the narrator reads aloud parts of a journal paper, was able to actively engage the listener—the subject—and thus induce awareness of the present moment, or what they were listening to. However, only the mindfulness exercise is able to evoke a new experience, because there are parts in the recording where the subjects are instructed to “take a mental trip around your body” and imagine new movements and sensations.

Moreover, the level of mindfulness was shown not to have an association with BCI performance in the UD task. Previous works have shown that the level of mindfulness is significantly correlated with SMR-based BCI performance in the UD task (Jiang et al., 2021b). Stieger et al. demonstrated that those that went through an 8-week MBSR training were better able to up-regulate the alpha power during intentional rest (Stieger et al., 2021b). Because intentional rest is the control signal to move the cursor downward within the UD task, a stronger alpha power would aid in classifying the up and down trials. Thus, we had initially surmised that the 20-min mindfulness intervention would have similarly positive effects. However, in this study, Spearman’s correlation analysis revealed no correlation between the total TMS Score (or either subscale within the TMS) and LR PVC, UD PVC, or the change in PVC for either task.

We also looked into whether the subjects were indeed meditating when they were instructed to do so. While there are known EEG patterns that may indicate whether or not a person is engaging in meditation, such as having increased theta and alpha bands (Takahashi et al., 2005; Lagopoulos et al., 2009; Ahani et al., 2013), it is extremely difficult to spot these patterns by eye, especially in real-time (online). Therefore, many attempts at classifying meditation versus non-meditation using various ML techniques also occur offline (Ren and Wu, 2014; Lu et al., 2017; Schirrmeister et al., 2017; Lawhern et al., 2018; Luo et al., 2018; Ma et al., 2018; Wang et al., 2018; Stieger et al., 2021a). Likewise, we also attempted to analyze the differences in EEG power during interventions and resting state offline. As shown in Figure 2, we see strong alpha band activities in the occipital region of the brain during meditation and control, likely because the subjects were asked to keep their eyes closed throughout the interventions (Teplan, 2002; Bazanova and Vernon, 2014). Conversely, the resting state EEG shows minimal alpha power in the same region, likely due to the subjects having been asked to keep their eyes open (Bazanova and Vernon, 2014). We believe that the relatively higher delta power in the frontal region during rest compared to meditation and control stems from the incomplete removal of artifacts related to eye blinks, despite IC removal during data pre-processing.

The second part of the study was to determine whether this short mindfulness exercise affects BCI performance. We found that neither intervention type showed any significant effect on both BCI tasks, LR or UD. Although there was a small numerical increase in the average LR and UD PVC in the post-intervention BCI runs compared to the pre-intervention runs for the meditators in session 1, the effect is not significant. Additionally, the control subjects also saw an increase in UD PVC following their intervention. Hence, the increase in UD PVC for the meditators could be due to partially attributable to learning, as it has been shown that BCI learning is possible not only between sessions but within one session as well (Meng et al., 2017; Jiang et al., 2021b). Considering within-session learning—as presented by the control group—the improvement in BCI performance following mindfulness exercise intervention is diminished. One possible explanation is that there is an element of fatigue. Prolonged execution of MI is known to induce mental fatigue and thus negatively impact MI EEG separability (Talukdar et al., 2019). Talukdar et al. reported that subjects started feeling fatigued during the 4th run, or between 36 and 48 min of MI, and by the 5th run that every subject felt fatigued. Since our study lasted anywhere from approximately 40 min to sometimes even beyond 50 min of MI per session, many subjects may have become fatigued. This would have affected their post-intervention BCI performance, especially the UD task since it was performed last.

Moreover, this modest performance improvement is not repeated in session 2. Another possible explanation is that the influence of meditation is far weaker compared to the variation in individual performance, or that it impacts certain individuals selectively. For example, the largest change in PVC in either direction in session 1 was +29.3 and −32%. Even if there were a slight improvement in PVC due to the brief meditation, these large variations in performance or even the potential selectiveness of the impact of meditation may have masked it.

Interestingly, Figure 4 shows a somewhat increasing trend between the change in BCI performance (PVC, %) and the interval between the two sessions (days). However, further studies need to be conducted to corroborate this finding, since this result was not based on a rigorously designed study protocol (the interval between sessions was not evenly distributed among the subjects), as it is not the main purpose of this study, and the sample size is relatively small (*n* = 10).

Lastly, the △control signal between the left and right trials of the LR and up and down trials of the UD tasks calculated using the EEG data during MI was shown to be significantly correlated to BCI performance (Figure 5). This demonstrates a valid offline approach to mimic the control signal in online BCI tasks. The △control signal did not significantly differ between the two tasks (LR or UD) or between the two subject groups (meditation or control). This agrees with the general EEG pattern that showed no significant difference between meditators and control subjects, and also with the behavioral data that showed no significant difference in BCI performance between the two groups of subjects.

One limitation of this study is that the experimental setup may have caused some subjects to become drowsy. The experimenters orally requested the subjects to stay attentive during the experiment and again immediately before the intervention started. However, the subjects were seated in comfortable positions with their eyes closed in a quiet room while listening to audio recordings, which may have created an environment for them to easily become drowsy. Drowsiness could have caused some subjects to miss out on the meditation intervention or even perform worse in later trials. Another limitation of this study is that the order in which the subjects performed MI was not randomized. Prolonged practice of MI is known to induce mental fatigue and impact MI EEG class separability (Talukdar et al., 2019). Therefore, we suspect that the subjects may have felt more fatigued toward the end of each session, when they were performing the UD tasks. Thus, fatigue would potentially compromise the positive effect that meditation may have had on the UD task. A third limitation is that the specific meditation intervention used in this study focused on imagery of movement. For example, the narrator tells the listener to imagine what it would feel like to move certain parts of the body. Since this recording of mindfulness meditation was not able to elicit a meditative state—as verified by the lack of difference in the mindfulness survey scores and EEG patterns—whether a recording of a different type of meditation can improve the level of mindfulness, improve SMR-based BCI performance, or both, warrants further study.

## Conclusion

In this study, we investigated the immediate effects of a short, 20-min meditation exercise on a user’s ability to control an SMR-based BCI system. Results show that the brief meditation exercise did not manifest significantly different EEG patterns compared to a control scheme. This is also illustrated in the behavioral data, with the level of mindfulness and BCI performance maintaining similar levels between the two groups of subjects. The lack of significant improvements in BCI performance after 20 min of meditation suggests that potentially longer meditation interventions are required to elicit observable improvements in controlling an SMR-based BCI.

## Data availability statement

The data supporting the conclusion are presented in the manuscript. Experimental EEG data can be downloaded from: https://doi.org/10.6084/m9.figshare.21644429.

## Ethics statement

The studies involving human participants were reviewed and approved by Institutional Review Board of Carnegie Mellon University. The patients/participants provided their written informed consent to participate in this study.

## Author contributions

JK was involved in study design, data collection, data analysis, and manuscript writeup. XJ was involved in study design, data analysis, and manuscript revision. DF was involved in study design, subject recruitment, data collection, and manuscript revision. YL and NA were involved with data collection. CG was involved in study design and manuscript revision. BH was involved in the conception, study design, supervision, and manuscript revision. All authors contributed to the article and approved the submitted version.
